# Real-Time Analysis of Imatinib- and Dasatinib-Induced Effects on Chronic Myelogenous Leukemia Cell Interaction with Fibronectin

**DOI:** 10.1371/journal.pone.0107367

**Published:** 2014-09-08

**Authors:** Adam Obr, Pavla Röselová, Dana Grebeňová, Kateřina Kuželová

**Affiliations:** 1 Department of Cellular Biochemistry, Institute of Hematology and Blood Transfusion, Prague, Czech Republic; 2 Department of Cell Biology, Faculty of Science, Charles University in Prague, Prague, Czech Republic; Institute of Molecular Genetics IMG-CNR, Italy

## Abstract

Attachment of stem leukemic cells to the bone marrow extracellular matrix increases their resistance to chemotherapy and contributes to the disease persistence. In chronic myelogenous leukemia (CML), the activity of the fusion BCR-ABL kinase affects adhesion signaling. Using real-time monitoring of microimpedance, we studied in detail the kinetics of interaction of human CML cells (JURL-MK1, MOLM-7) and of control BCR-ABL-negative leukemia cells (HEL, JURKAT) with fibronectin-coated surface. The effect of two clinically used kinase inhibitors, imatinib (a relatively specific c-ABL inhibitor) and dasatinib (dual ABL/SRC family kinase inhibitor), on cell binding to fibronectin is described. Both imatinib and low-dose (several nM) dasatinib reinforced CML cell interaction with fibronectin while no significant change was induced in BCR-ABL-negative cells. On the other hand, clinically relevant doses of dasatinib (100 nM) had almost no effect in CML cells. The efficiency of the inhibitors in blocking the activity of BCR-ABL and SRC-family kinases was assessed from the extent of phosphorylation at autophosphorylation sites. In both CML cell lines, SRC kinases were found to be transactivated by BCR-ABL. In the intracellular context, EC50 for BCR-ABL inhibition was in subnanomolar range for dasatinib and in submicromolar one for imatinib. EC50 for direct inhibition of LYN kinase was found to be about 20 nM for dasatinib and more than 10 µM for imatinib. Cells pretreated with 100 nM dasatinib were still able to bind to fibronectin and SRC kinases are thus not necessary for the formation of cell-matrix contacts. However, a minimal activity of SRC kinases might be required to mediate the increase in cell adhesivity induced by BCR-ABL inhibition. Indeed, active (autophosphorylated) LYN was found to localize in cell adhesive structures which were visualized using interference reflection microscopy.

## Introduction

Hematopoietic cell interaction with the extracellular matrix of the bone marrow influences the cell behaviour and development. The microenvironment regulates e.g. cell division rate, resistance to apoptosis and cell differentiation [1]. Chronic myelogenous leukemia (CML), which is characterized by the presence of the fusion tyrosine kinase BCR-ABL, is associated with altered cell interaction with extracellular matrix proteins [2], [3]. In the advanced phases of CML, leukemic progenitors are prematurily released into the blood stream, probably due to decreased cell adhesivity to the bone marrow and to an altered response to chemokines, such as SDF-1. Since the introduction of targeted therapy using tyrosine kinase inhibitors, the majority of patients remain in long-term complete remission. However, the discontinuation of the therapy usually leads to disease relapse which indicates that the residual disease remains to be a major issue in CML management [4]. Intense research in this field indicates that the leukemic burden arises from quiescent, non-dividing cells which are resistant to treatment [5], [6]. Understanding the mechanisms regulating cell quiescence, such as cell adhesion to the bone marrow matrix, is thus important for further progress in CML therapy.

We have recently described a novel approach to monitor hematopoietic cell interaction with selected extracellular matrix proteins. Real-time measurement of microimpedance allows for monitoring changes in cell adhesion to surfaces coated with the protein of interest. In this work, we applied this method to study the interaction of CML-derived cell lines with fibronectin and the effects of the most commonly used tyrosine kinase inhibitors, imatinib (mainly targeting BCR-ABL) and dasatinib (a dual ABL/SRC family kinase inhibitor).

## Materials and Methods

### Chemicals

Dasatinib was purchased from Selleckchem, 50 mM and 1 mM stock solutions were made in sterile dimethylsulfoxide. Imatinib was obtained from Novartis (Basel, Switzerland), 2 mM stock solution was prepared in sterile water. Fibronectin fragment (120 kDa cell attachment region) was purchased from Chemicon International (CA, U.S.A.). To prepare a fibronectin-coated plate, 50 µl of fibronectin fragment solution (20 µg/ml in sterile water) was added to each well of a Nunc Maxisorp 96-well microtitration plate or of a 16-well E-plate used for real-time cell adhesion monitoring. The plates were subsequently incubated overnight at 10°C. After incubation, the plates were washed three times in PBS and blocked in 1% bovine serum albumin (BSA) in PBS (200 µl/well, 30 min at room temperature). The plate was washed in PBS once again immediately before use.

Antibodies against phospho-SRC (Y417) family (#2101) and phospho-c-ABL (Y245, #2861) were purchased from Cell Signaling. Antibodies against HCK (#610278) and LYN (#610003) were purchased from BD Biosciences, antibody against LCK (Y123, #ab32149) was purchased from Abcam.

### Cell isolation and culture

JURL-MK1 cell line was purchased from DSMZ (German Collection of Microorganisms and Cell Cultures, Braunschweig, Germany), JURKAT cell line was from European Collection of Animal Cell Cultures (Salisbury, UK). MOLM-7 and HEL cells were provided by J. Minowada [7] and P. Martin [8], respectively. Cell lines were cultured in RPMI 1640 medium supplemented with 10% fetal calf serum, 100 U/ml penicillin and 100 µg/ml streptomycin at 37°C in 5% CO_2_ humidified atmosphere.

Primary blood cells were obtained from leukapheresis product from patients with chronic myelogenous leukemia. Peripheral blood mononuclear cells (PBMC) were separated by standard density gradient centrifugation using Histopaque-1077 (Sigma) and maintained in RPMI 1640 medium described above.

### Ethics statement

Primary leukemic blood cells were isolated from leukapheresis products, following written informed consent of the patient as to the use of biological material for research purposes. The research was approved by the Ethics Committee of the Institute of Hematology and Blood Transfusion.

### Measurement of cell adhesivity to fibronectin

The end-point method for assessment of cellular adhesivity to fibronectin-coated surface has been descibed previously [9]. Briefly, the cells (1×10^4^) were seeded into fibronectin-coated wells on a microtitration plate and incubated for 1 h at 37°C. Then, the cells were washed wih PBS/Ca^2+^/Mg^2+^ and the remaining cells were quantified by means of fluorescent labelling (CyQuant Cell Proliferation Assay Kit; Molecular Probes). The adherent cell fraction (ACF) was calculated using the fluorescence signal from fibronectin-coated plate and the signal obtained from a reference plate that contained the total cell number.

### Real-time microimpedance measurement

The real-time cell analysis was performed as described previously [10]. RTCA xCELLigence DP system (Acea Biosciences, San Diego, CA, U.S.A.) was placed in an incubator (at 37°C) with 5% CO_2_ content. Fibronectin-coated E-plates were filled with 100 µl of RPMI 1640 and equilibrated at 37°C. The microimpedance signal (cell index) was set to zero in these conditions. The cells (60000/well) were added in 100 µl of suspension in RPMI 1640. Imatinib was pre-diluted in RPMI 1640 and added in total volume of 2 µl/well in triplicates or quadruplicates, respectively. Dasatinib was pre-diluted in sterile DMSO and added in total volume of 0.2 µl/well in triplicates or quadruplicates. The final DMSO concentration in wells did not exceed 0.1%. DMSO (0.1%) was also added to control wells.

### Immunoblotting

SDS-polyacrylamide gel electrophoresis was performed as described before [11]. Briefly, the cells (5×10^6^) were lysed in modified RIPA buffer and 15 µg of total protein (30 µg for BCR-ABL detection) was resolved with SDS-PAGE and transferred for 1 h (3 h for BCR-ABL) on a nitrocellulose membrane. Proteins of interest were detected using the appropriate antibodies and the chemiluminiscent signal was visualised and evaluated using G-BOX iChemi XT4. To determine EC50 values, relative band intensities at different inhibitor concentrations were fitted with sigmoidal curves using GraphPad Prism 5 software.

### Flow cytometry

The cells (1×10^6^) were washed once in PBS, fixed and permeabilized with FIX&PERM Cell Permeabilization Kit (An Der Grub, Kaumberg, Austria), and incubated with anti-phospho-SRC family antibody overnight at 4°C and then with anti-rabbit PE-conjugated secondary antibody for 1 h at room temperature. Detection of PE signal was performed using LSRFortessa flow cytometer (BD Biosciences).

### Immunofluorescence microscopy

The cells were plated on fibronectin coated coverslips or glass bottom dishes, incubated for 60 minutes in CO_2_ incubator, fixed with 2% paraformaldehyde, permeabilized in 0.3% Triton/PBS and incubated with anti-phospho-SRC family primary antibody and a secondary antibody (Alexa Fluor 488 Goat Anti-Rabbit). F-actin was labeled with rhodamine phalloidin (Molecular Probes). Samples were analyzed using an Olympus Fluoview FV1000 confocal laser scanning microscope.

### Interference reflection microscopy

Cells were incubated for 1 h on fibronectin-coated coverslip and fixed with 2% paraformaldehyde. The interference in reflected light was observed by means of FV-1000 confocal microscope (Olympus), using 405 nm laser beam and focusing to the coated glass surface.

## Results

**Figure 1:**
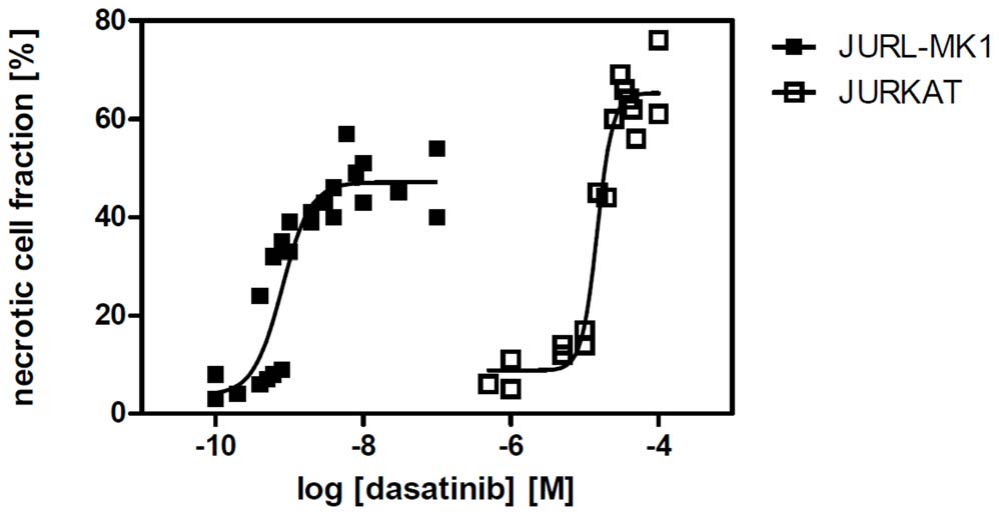
Dasatinib-induced cell death in BCR-ABL-positive and -negative cell lines. JURL-MK1 (full squares) and JURKAT (empty squares) cells were treated with dasatinib at different concentrations as indicated. After 45–48 h incubation, the fraction of non-viable cells was determined by counting of trypan blue-stained samples. Summary of 3 independent experiments for each cell line is shown.

We analyzed the effects of imatinib mesylate and dasatinib on two CML-derived cell lines (JURL-MK1 and MOLM-7), and two cell lines not expressing BCR-ABL protein (HEL and JURKAT). As expected, both tyrosine kinase inhibitors (TKIs) reduced cell growth and induced cell death in BCR-ABL-positive cells while they were considerably less toxic in BCR-ABL-negative cells. Fig. 1 illustrates the difference in dasatinib toxicity between JURL-MK1 and JURKAT cells. EC50 values for cell growth inhibition and for cell death induction in all four cell lines are given in Table 1.

**Table 1:** EC50 values for effects induced by imatinib and dasatinib.

cell line:	imatinib EC50	dasatinib EC50
JURL-MK1	proliferation (0.12±0.05) µM	proliferation (0.7±0.1) nM
	cell death (0.35±0.12) µM	cell death (0.8±0.3) nM
MOLM-7	proliferation (0.34±0.04) µM	proliferation (1.0±0.4) nM
	cell death (1.8±0.8) µM	cell death (2.1±1.0) nM
HEL	>10 µM	proliferation (15±1) µM
		cell death (39±6) µM
JURKAT	>10 µM	proliferation (11±3) µM
		cell death (21±4) µM

Cells were treated with the inhibitors at different concentrations for 48 h and counted. Cell viability was assessed using Trypan blue exclusion test. Measured values were fitted by sigmoidal curves using GraphPad Prism 5.0 software. Given EC50 values for inhibition of cell growth and for cell death induction are means and standard deviations from at least 3 independent experiments.

We have previously shown that imatinib mesylate affected JURL-MK1 cell adhesivity to fibronectin (FN), one of the major components of the extracellular matrix [12]. During first several hours of imatinib treatment, the cell fraction adhering to FN increased. On the contrary, longer imatinib treatment resulted in a reduction of cell binding to FN, probably due to the onset of apoptosis. These results suggested that BCR-ABL inhibition strengthens JURL-MK1 cell interaction with FN. Similar results were also obtained using dasatinib (Figure S1). After about 6 h of dasatinib treatment, but not before, caspases became activated and apoptotic cells started to appear (Figure S1, panels A–C). Following 24 h treatment, the apoptosis was triggered in a large cell fraction and, accordingly, cell adhesivity to fibronectin lowered. This decrease was prevented by simultaneous addition of the caspase inhibitor Q-VD-OPh (Figure S1, panel D). We also performed standard Annexin V staining following 6 h treatment of JURL-MK1 and MOLM-7 cells with 10 nM dasatinib and found no significant increase in the number of early apoptotic cells at this time point.

**Figure 2:**
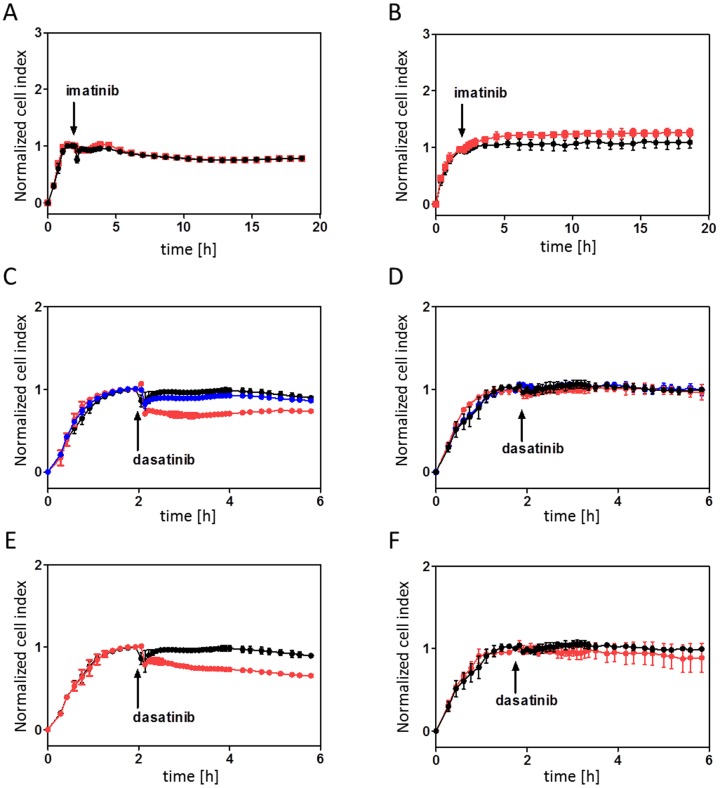
Changes in cell interaction with fibronectin after inhibitor treatment. The cells (6×10^4^ per well) were seeded into fibronectin-coated E-plates. After the microimpedance signal stabilization, the appropriate inhibitor was added in triplets. Black circles: control cells. Time of inhibitor addition is indicated by an arrow. Microimpedance signal (cell index) was normalized to 1 at the time of inhibitor addition. The graphs show mean and standard deviation of well triplets. A,C,E: JURL-MK1 cells, B,D,F: MOLM-7 cells. A,B: imatinib was added at 1 µM (blue circles) or 10 µM (red squares) final concentration. C,D: dasatinib was added at 2 nM (blue circles) or 10 nM (red squares) final concentration. E,F: dasatinib was added at 100 nM final concentration (red circles).

In this work, we studied the kinetics of cell-FN interaction after TKI treatment in detail using a recently introduced method based on monitoring of microimpedance between two interdigitated microelectrode systems embedded in the bottom of the wells of a microtitration plate [10]. We showed that, in case of hematopoietic cells, the microimpedance signal (denoted as “cell index”) reflects cell interaction with FN-coated surface. Figure 2 shows representative examples of the observed kinetics for the two CML cell lines, JURL-MK1 and MOLM-7. Cells were seeded into E-plates and incubated for 1–2 h to allow for cell attachment to the coated cell bottom. After signal stabilization, the drug was added in a small drop while the corresponding solvent was added to control wells as any mechanical perturbation usually produced minor changes, too. Imatinib mesylate addition to BCR-ABL-positive cells resulted in nearly immediate increase in the microimpedance signal, the effect being enhanced at higher imatinib concentration (Figure 2A,B). On the other hand, no appreciable change occurred in the control BCR-ABL-negative cell lines (Figure S2). More complex behaviour was observed after dasatinib treatment: at low (about 0.5 to 10 nM) concentration, dasatinib effect was similar to that of imatinib (Figure 2C,D). With increasing dasatinib dose, the effect was gradually suppressed and disappeared at several tens of nM (Figure 2E,F). Furthemore, at very high dasatinib concentration (up from 1 µM), an increase in the signal was observed in CML-derived cell lines as well as in BCR-ABL-negative cell lines (Figure S3) and even in freshly isolated primary mononuclear cells.

**Figure 3:**
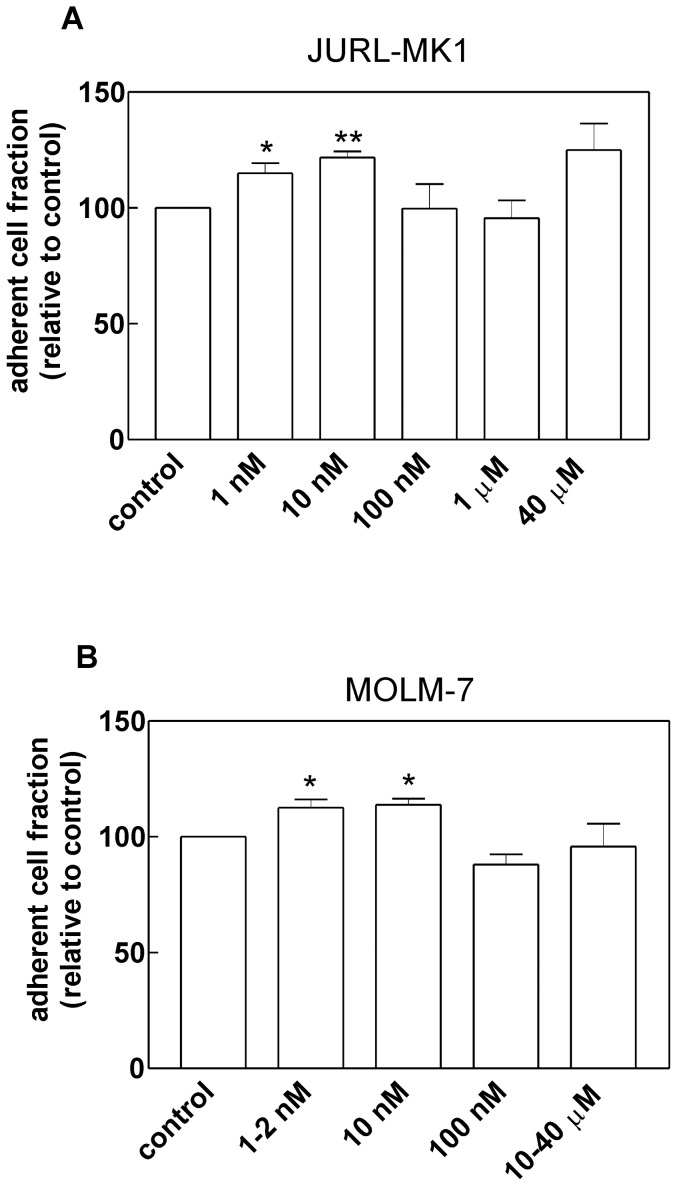
Changes in the adhered cell fraction induced by dasatinib pretreatment. JURL-MK1 (A) or MOLM-7 (B) cells were pretreated for 30 min with dasatinib at the indicated concentrations, seeded into FN-coated wells and incubated for 1 h at 37°C. Thereafter, the plate was washed and the fraction of attached cells was determined fluorimetrically and normalized using the value found in the control sample. The graphs show the means and standard deviations from at least 4 experiments for each condition, the statistical significance of the observed differences was evaluated using Student's paired t-test. Results significantly differing from untreated controls are denoted by asterisks (* p<0.05, ** p<0.01).

The increase in the microimpedance signal may be due to an increase in the fraction of interacting cells, but also to an increase in the individual cell area in contact with the coated surface (cell spreading) or to closer cell nestling against the surface. We thus compared the fraction of cells attached to FN-coated wells for control and dasatinib-treated cells. The cells were either seeded into the coated plate and subsequently treated with dasatinib or pretreated for 30 min with the drug and seeded thereafter. In both experimental settings, the results were similar. Both in JURL-MK1 and MOLM-7 cells, low dasatinib concentrations (1 to 10 nM), but not the intermediate ones (100 nM-1 µM), induced a mild increase in the number of attached cells (Figure 3). However, the changes were less pronounced in comparison with those seen in microimpedance measurements (Figure 2C,D).

**Figure 4:**
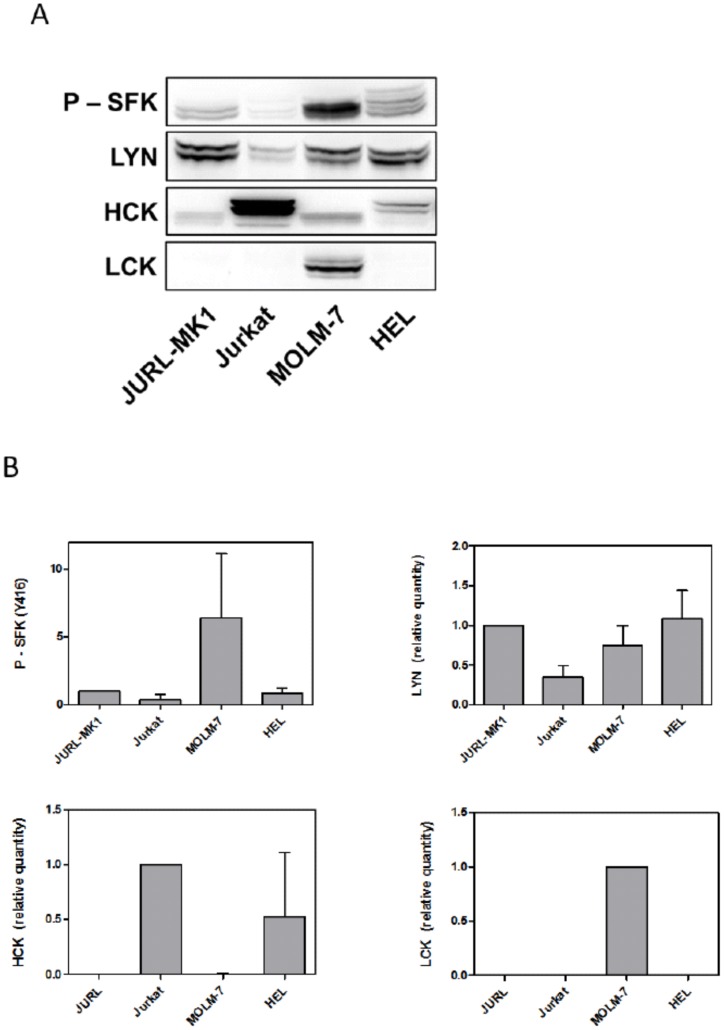
SRC family kinase profiles of leukemic cell lines. A: p-Y416 SFK, LYN, HCK and LCK expression pattern in the used cell lines. B: Summary of relative expression levels of p-Y416 SFK, LYN, HCK and LCK kinases in cell lines. Several (2–5) western-blots were quantified and normalized to the signal of JURL-MK1 cell line (p-SFK, LYN), JURKAT cell line (HCK) or MOLM-7 cell line (LCK).

To account for possible roles of BCR-ABL and SRC-family kinases (SFKs) in the observed adhesivity changes, we analyzed the extent and the kinetics of inhibition of these kinases due to TKI treatment. First, we screened the studied cell lines for mRNA transcripts of the individual members of SRC kinase family that are known to be expressed in hematopoietic cells (LYN A, LYN B, HCK, LCK, FGR). We found that all the four cell lines express mRNA for two Lyn splicing variants, LynA and LynB. In addition, Hck and Lck mRNA, but not Fgr, were expressed in some of them. The protein products of the relevant SFK genes were subsequently quantified by means of western-blotting (Figure 4). SFKs contain several autophosphorylation sites which may be used to assess the activation status of these kinases. The phospho-specific antibody we used recognizes Tyr 416 in c-SRC and the corresponding residues in HCK, LYN and LCK. LYN A and B are detected at 56 and 53 kDa, respectively, LCK at 54–55 kDa and two HCK isoforms (produced using different sites of initiation of translation) at 59 and 61 kDa.

To detect the activated form of BCR-ABL protein, phospho-specific antibody against phosphorylated Tyr245 of c-ABL was used and the band at 210 kDa corresponding to BCR-ABL was quantified.

**Figure 5:**
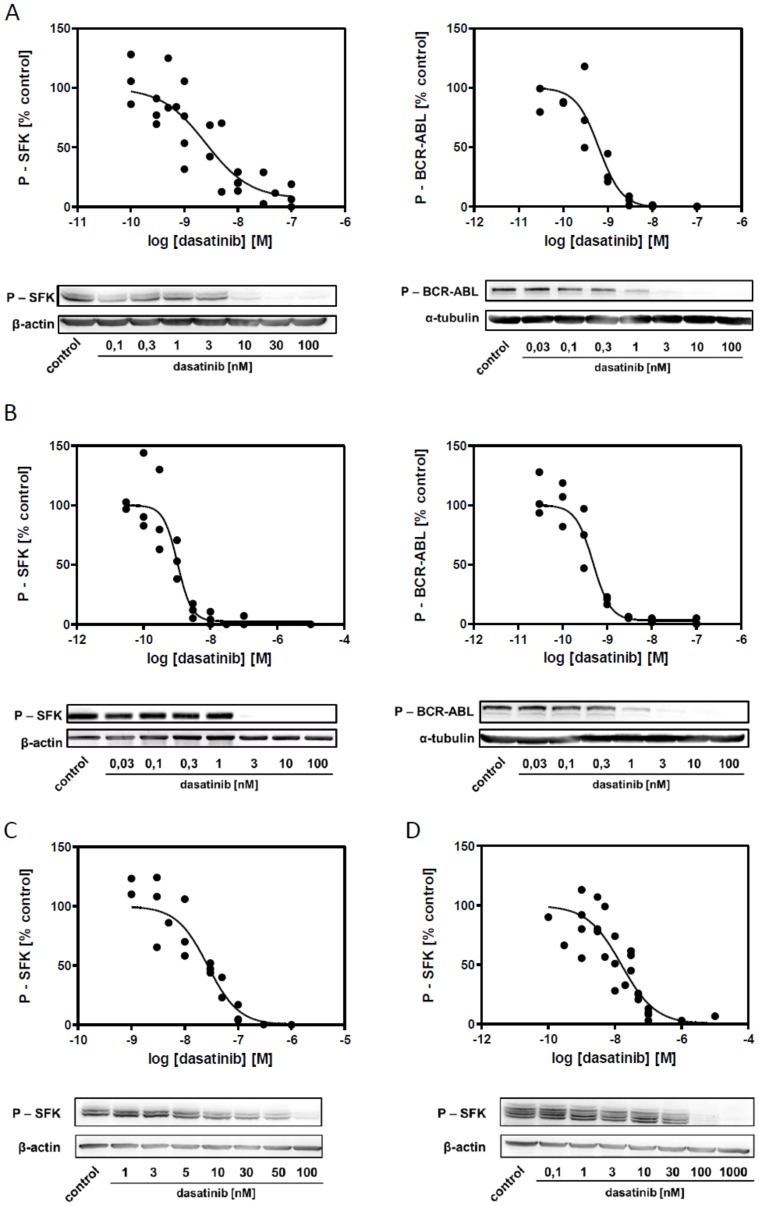
Dasatinib-induced dephosphorylation of SFK and BCR-ABL. Phosphorylation status of SFK and BCR-ABL after 2 h incubation with dasatinib. Quantification summary from 3 independent experiments and representative western-blots are shown for each cell line. A: JURL-MK1, B: MOLM-7, C: HEL, D: JURKAT.

The decrease in levels of phosphorylated BCR-ABL and SFKs after 2 h incubation with dasatinib at different concentration is documented in Figure 5 and EC50 values for BCR-ABL and SFK inhibition obtained from repeated experiments are given in Table 2. Note that similar results were obtained when the levels of phosphorylated SFKs were analyzed using flow-cytometry instead of western-blotting (Figure S4). We have also performed similar experiments with primary mononuclear cells from CML patients in the chronic phase of the disease (N = 3) and EC50 values for SFK dephosphorylation after 2 h dasatinib treatment ranged between 20 and 29 nM.

**Table 2:** EC50 values for dasatinib-induced dephosphorylation of BCR-ABL and SFKs.

cell line:	BCR-ABL (nM)	SFK (nM)
JURL-MK1	**0.6** *(0.4–1.0)*	**2.5** *(1.0–6.2)*
MOLM-7	**0.5** *(0.3–0.7)*	**1.0** *(0.7–1.6)*
HEL	-	**27** *(15–48)*
JURKAT	-	**15** *(6–41)*
		HCK **5.2** *(1.8–15)*
		LYN **22** *(9–53)*

Cells were incubated for 2 h with dasatinib at different concentrations and the levels of phosphorylated kinases were then determined by western-blotting using phospho-specific antibodies. Relative values (% of controls) from at least 3 independent experiments were merged and the summary results were fitted with sigmoidal dose-response curves using GraphPad Prism 5.0 software. The table shows the obtained EC50 values along with 95% confidence interval. In case of JURKAT cells, bands corresponding to p-HCK and p-LYN were also evaluated separately.

**Figure 6:**
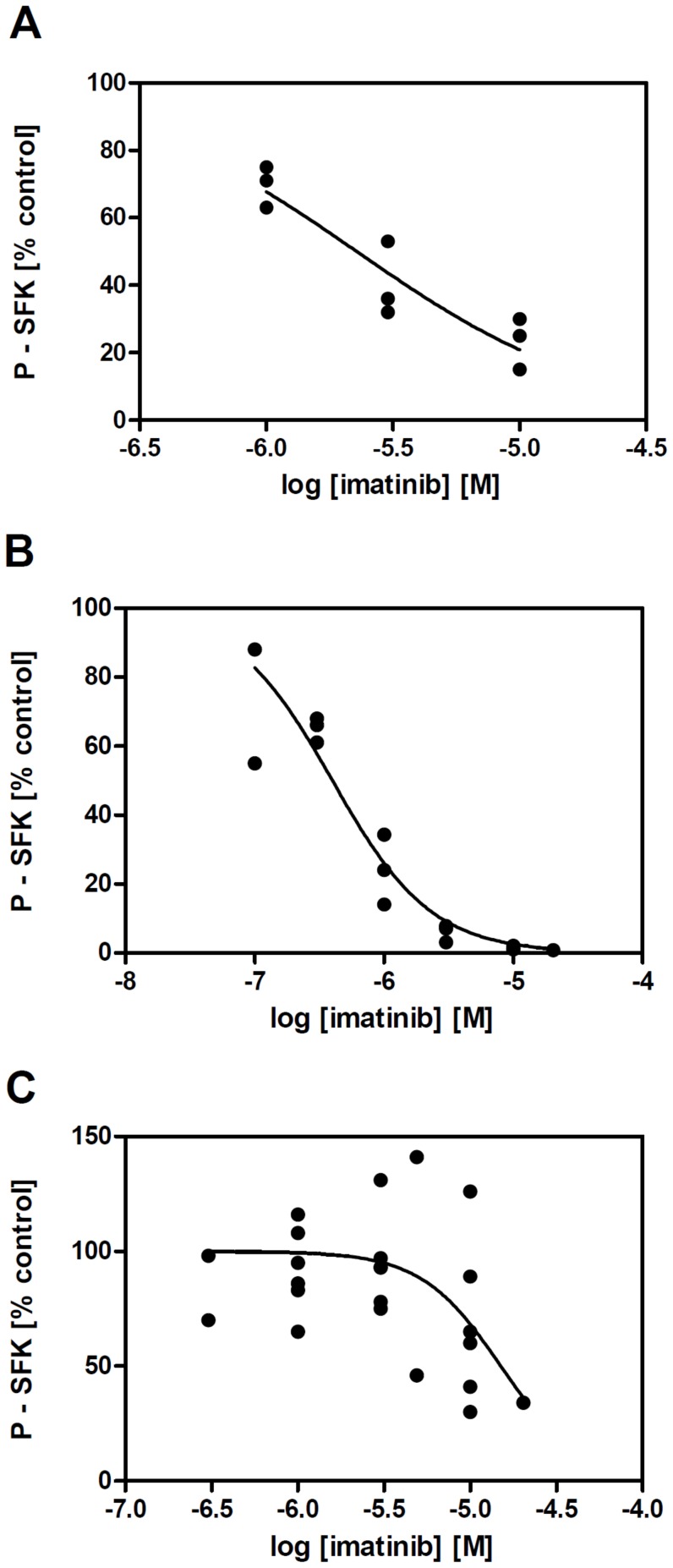
Imatinib-induced dephosphorylation of SFK in BCR-ABL-positive and -negative cell lines. Phosphorylation status of SFK after 2 h incubation with imatinib was assessed by western-blotting. Summary of independent experiments for JURL- MK1 (A), MOLM-7 (B) and HEL cells (C).

When comparing EC50 values for dasatinib-mediated SFK inhibition in BCR-ABL-positive vs BCR-ABL-negative cells, we noted a clear difference indicating that SFK dephosphorylation in BCR-ABL-positive cells might be due to an indirect effect. SFKs are known to be targets of BCR-ABL and inhibition of BCR-ABL can thus result in a decrease in SFK activity. We thus assessed SFK phosphorylation status after treatment with imatinib which is considered to be relatively specific for BCR-ABL inhibition. Indeed, 1 µM imatinib treatment markedly reduced SFK phosphorylation in BCR-ABL-positive cells while it had no or mild effect in HEL cells (Figure 6 and Figure S4).

**Figure 7:**
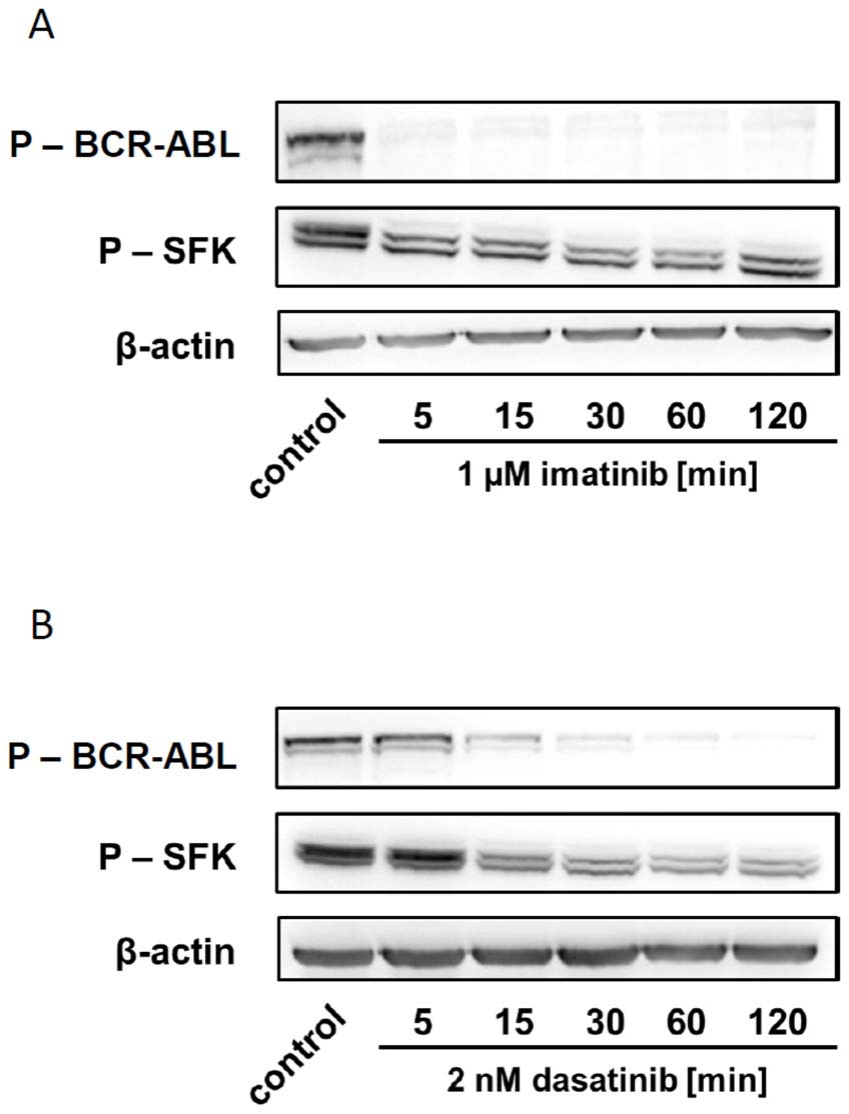
Time-course of BCR-ABL and SFK dephosphorylation after treatment with inhibitors. Time-resolved decrease in the phosphorylation status of BCR-ABL and SFK in JURL-MK1 cells after treatment with 1 µM imatinib (A) or 2 nM dasatnib (B). The experiment was repeated with closely similar results.

The time course of kinase dephosphorylation in JURL-MK1 cells is shown in Figure 7. The effect of imatinib addition was very rapid: BCR-ABL was dephosphorylated in 5 minutes and the activity of SFKs was reduced at the same time. The kinetics of dephosphorylation was somewhat slower for 2 nM dasatinib. However, the decrease of BCR-ABL and SFK phosphorylation also occurred simultaneously. As expected, 100 nM dasatinib was sufficient to rapidly inhibit virtually all SFK molecules.

**Figure 8:**
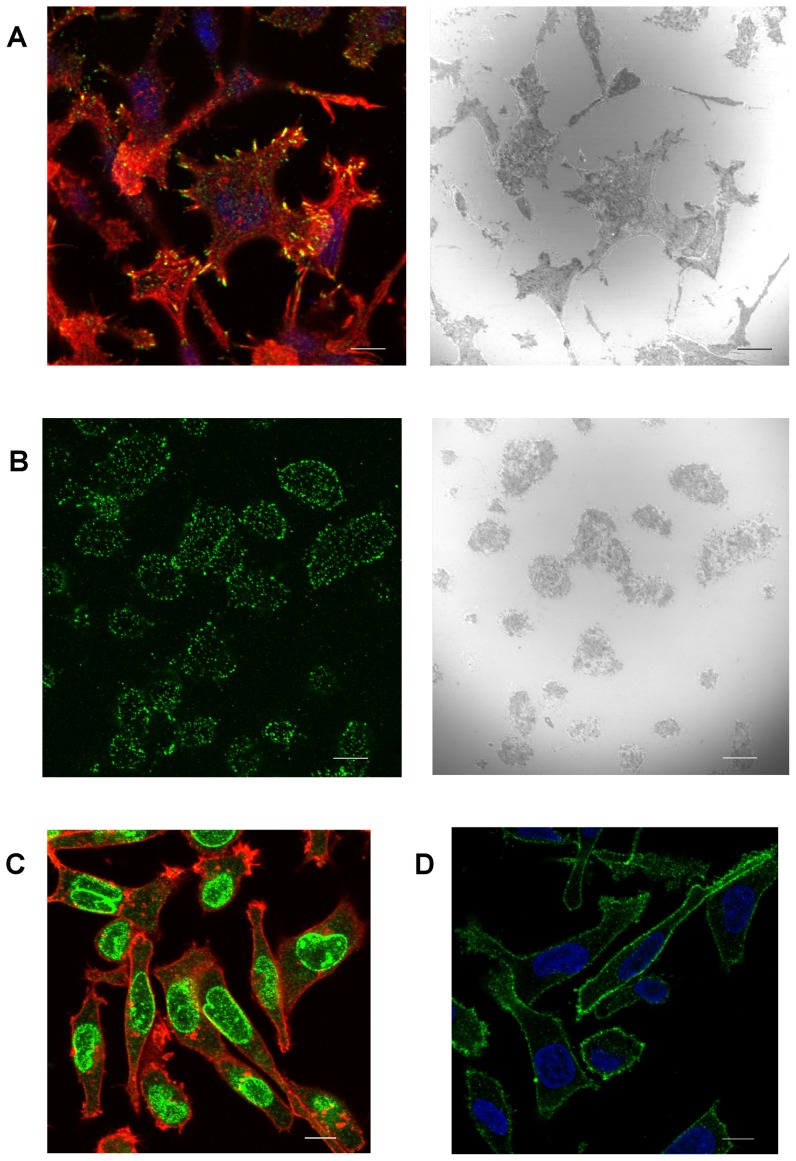
Microscopic analysis of SRC kinases localization in cells on fibronectin. MOLM-7 (A, C, D) or JURL-MK1 (B) cells were plated on fibronectin-coated slide, incubated for 1 h at 37°C, fixed and stained with antibodies against phospho-SFK (A, B), LCK (C) or LYN (D). A, B: Immunofluorescence images (left) were taken from the slice immediately adjacent to the FN-coated surface. Right images in A and B were captured in interference reflection mode. Green signal: SFK, red: actin (stained with phalloidin), blue: cell nuclei (DAPI). Scale bars: 10 µm.

To support our hypothesis that SFKs may be involved in regulation of cell interaction with fibronectin, we studied the intracellular localization of active SFKs using the phospho-specific anti-SFK antibody. MOLM-7 cells on fibronectin had a spread morphology with numerous marked membrane protrusions while JURL-MK1 cells had a more compact form. Figure 8 A,B (right panels) shows images obtained using the interference reflection microscopy which allows for visualization of cell areas that are near to the coated surface. Immunofluorescence staining clearly shows an accumulation of active SFKs at the ends of membrane protrusions and in other areas of cell-surface contacts in MOLM-7 cells (Figure 8A, left panel). In JURL-MK1 cells, the active SFKs were found mainly in the contours of cell-surface contact areas and their localization to the sites of the closest contact was less evident due to different morphology (Figure 8B). As expected, 100 nM dasatinib treatment resulted in complete disappearance of the green fluorescent signal from adhesion sites (Figure S5). The same effect was also reached using the most commonly used SFK inhibitor PP2 (Figure S5, right panels). As the main SFKs in JURL-MK1 and MOLM-7 were LYN and LYN+LCK, respectively, we also analyzed the intracellular localization of these individual kinases. LCK was mainly present in the nuclear membrane (green signal in Figure 8C) while LYN was predominantly found in the cytoplasmic membrane and in the cytoplasm (green signal in Figure 8D for MOLM-7 cells, similar staining pattern was observed in JURL-MK1 cells).

## Discussion

The analysis of cytotoxic effects of both tyrosine kinase inhibitors (Table 1) confirmed that these effects are related to the inhibition of BCR-ABL protein. EC50 values for JURL-MK1 and MOLM-7 cell death induction by imatinib are in agreement with results previously described for other cell lines [13]–[15] as well as with reported IC50 values for purified BCR-ABL protein (0.2–0.3 µM, [16], [17]). Similarly, toxic effects of dasatinib on BCR-ABL-positive cells occur in the concentration range corresponding to IC50 value of dasatinib towards the isolated BCR-ABL (0.8–1.3 nM, [16], [18], [19]) and to EC50 values for BCR-ABL dephosphorylation in the intracellular context (Table 2, [14], [15], [19]–[21]). On the other hand, inhibition of SRC kinases does not have toxic effects in BCR-ABL-negative cells. The induction of cell death at high dasatinib concentrations (more than 1 µM) is undoubtedly due to the inhibition of unspecific dasatinib targets.

Using real-time monitoring of microimpedance, we performed kinetic analysis of the effects of imatinib and dasatinib on hematopoietic cell interaction with fibronectin fragment as a simple model of the bone marrow extracellular matrix. It is known that c-ABL signaling affects the adhesion pathways at many points [22], and that BCR-ABL-transformed cells show abnormalities in adhesion processes [3], [23]–[26]. However, the resulting effect of BCR-ABL transformation on cell adhesion remains unclear, and seems to depend on cell type and/or used methodic approach. In a similar way, SFKs are involved in cell adhesion to the extracellular matrix in adherent cell types [27]–[29] and in mature hematopoietic cells, but the role of SFKs in hematopoietic progenitor cell adhesion signaling is still rather unclear.

Imatinib mesylate and low-dose dasatinib strengthened the interaction of JURL-MK1 and MOLM-7 cells with fibronectin (Figure 2 A–D). The effect occurred shortly after inhibitor addition and lasted for many hours. The subsequent decrease is likely to be related to the onset of apoptosis [12]. We have also detected drug-induced increase in the number of cells which are firmly bound to FN, although this effect was less pronounced (Figure 3). This indicates that an increase in cell spreading or closer cell contact with the coated surface contributes to the observed changes in microimpedance signal.

Both imatinib and low-dose dasatinib were found to reduce the phosphorylation (activity) of SFKs in BCR-ABL-positive cells. This effect is probably indirect, resulting from the loss of SFK stimulation by BCR-ABL [30], [31], as 1 µM imatinib does not significantly reduce SFK phosphorylation in BCR-ABL-negative HEL cells (Figure 6, Figure S4) although IC50 values for isolated LYN were reported to be 0.35–2.2 µM [17], [32], [33]. In addition, in our experiments, EC50 values for SFK inhibition by dasatinib in BCR-ABL negative cells are by one order of magnitude higher than those in BCR-ABL-positive cells (Table 2). EC50 of dasatinib for LYN inhibition in the intracellular context is of about 20 nM (Table 2, values for JURKAT and HEL cells), while reported IC50 values for recombinant enzyme inhibition range from 1.7 to 8.5 nM [16], [34], [35]. We have also recently reported that PP2, an inhibitor of SFKs, increases JURL-MK1 cell adhesivity to FN. However, we found later that PP2 reduces the activity of BCR-ABL as well, although it is not clear if the decrease in BCR-ABL phosphorylation is due to a loss of SFK-mediated stimulation or to direct inhibition (Figure S6). No really specific small molecule inhibitor is currently available and it is thus difficult to dissect the individual roles of BCR-ABL and SFKs in dynamic cell response to imatinib and dasatinib. Nevertheless, as the inhibitors induce no significant increase of the microimpedance signal from BCR-ABL-negative cells, it is likely that the effects of TKIs on CML cell adhesion are prevalently due to BCR-ABL inhibition.

The increase in CML cell adhesivity to FN was suppressed at higher dasatinib doses (e.g. 100 nM) corresponding to concentrations which are achieved in clinical settings [36]. This indicates that dasatinib at these concentrations targets a protein which is required for dynamic cell interaction with fibronectin. We speculate that such target could be represented by SFKs as suggested by EC50 values for their inhibition by dasatinib (Table 2). Indeed, SFKs are important regulators of adhesion signaling in adherent cells. The prototypic member of the family, c-SRC, belongs to well-known oncogenes. In physiological conditions, c-SRC is maintained inactive and is activated only transiently. Sustained increased activity of c-SRC shifts the dominance in adhesion signalization towards RAC1/CDC42 axis instead of RHOA and cells transformed with c-SRC acquire migratory phenotype including anchorage-independent growth and loss of contact inhibition. The other members of SRC family are much less explored, especially in relation to the cell adhesivity. Some of them (LYN, HCK, FGR, LCK, BLK) are more or less specifically expressed in hematopoietic cells where they are required for normal hematopoiesis. The current model describes SFKs as rheostats in immune cell signaling, with positive or negative net response being dependent on a number of stimulatory conditions [37]. Major SFKs expressed in myeloid cells, LYN and HCK, seem to have mutually opposed functions in integrin-mediated signaling in mature myeloid cells. In neutrophils and macrophages, LYN acts as a negative regulator of integrin-mediated adhesion, as LYN −/− cells show hyper-adhesive phenotype [38]. On the other hand, HCK and FGR −/− macrophages and neutrophils show disruption of integrin-mediated signaling, suggesting a positive role for HCK and FGR kinases in integrin-mediated adhesion [39], [40]. Neutrophils deficient in HCK and FGR also display defects in migration processes [41]. In immature hematopoietic precursors, downregulation of LYN expression using siRNA resulted in higher adhesion to stroma and reduced CXCR4-dependent chemotaxis [42], which is needed for hematopoietic cell homing to the bone marrow [6].

In our cell lines, LYN was omnipresent while HCK and LCK were found in some of them only (Figure 4). MOLM-7 cells express LYN and LCK, the latter being localized mainly to the nuclear membrane (Figure 8C). In keeping with previously reported results obtained in other cells [43], LYN was predominantly present in the cytoplasmic membrane and, to a lesser extent, in the cytoplasm (Figure 8D). Importantly, immunofluorescence staining showed that active SFKs accumulate in adhesion structures formed at MOLM-7 cell contacts with FN-coated surface, especially at the extremities of prominent membrane protrusions (Figure 8A). This would support the hypothesis that hematopoietic SRC kinases, and especially LYN, are involved in rearrangement of adhesion structures and that a minimal LYN activity is required for BCR-ABL-induced changes in cell interaction with fibronectin. On the other hand, SFKs are clearly not required to the establishment of cell contacts with FN as the cells pretreated with high-dose dasatinib were still able to attach to FN-coated surface (Figure 3).

In any case, it appears from our experiments that dasatinib at therapeutic concentrations does not enhance CML cell binding to fibronectin (Figures 2 E,F and 3). It is not known if this is also true for primary leukemic blasts and their interaction with relevant extracellular matrix proteins. However, if so, dasatinib-based therapy might lead to more efficient eradication of the residual disease in comparison with imatinib treatment which could enhance the protective effect provided by the bone marrow microenvironment to leukemic blasts.

The changes observed at very high dasatinib concentrations (more than 1 µM, Figure S3) are not relevant for the clinical practise. Nevertheless, these results suggest that dasatinib at these concentrations targets a protein which is important for hematopoietic cell adhesion signaling in general, as these effects were observed in all the studied cell lines as well as in primary mononuclear cells. Therefore, uncovering of this target will be the subject of a subsequent proteomic study.

## Supporting Information

Figure S1 **Kinetics of dasatinib-induced cell death and changes in cell adhesivity to fibronectin.** A: Apoptosis induction in JURL-MK1 and MOLM-7 cells during treatment with 10 nM dasatinib was assessed by flow-cytometry. Cell fraction in sub-G1 region of cell cycle phase distribution in samples stained with propidium iodide. B: Caspase activation during cell incubation with 10 nM dasatinib was monitored using fluorogenic substrate Ac-DEVD-AFC following the same protocol as in the reference 12. C: Caspase activation in cells treated with 1 to 100 nM dasatinib for 2 or 6 h (same method as in B). D: Kinetics of changes in JURL-MK1 cell adhesivity to fibronectin following treatment with 2 nM dasatinib alone or in combination with 10 µM Q-VD-OPh (method as in Fig. 3).
(PDF)

Figure S2 **Changes in cell interaction with fibronectin after imatinib treatment.** The cells (6×10^4^ per well) were seeded into fibronectin-coated E-plates. After the microimpedance signal stabilization, the appropriate inhibitor was added in triplets. Black circles: control cells. Time of inhibitor addition is indicated by an arrow. Microimpedance signal (cell index) was normalized to 1 at the time of inhibitor addition. The graphs show means and standard deviations of well triplets. A,C,E: HEL cells, B,D,F: JURKAT cells. A,B: imatinib was added at 10 µM (red squares) final concentration. C,D: dasatinib was added at 2 nM (blue circles) or 10 nM (red squares) final concentration. E,F: dasatinib was added at 100 nM final concentration (red circles).
(PPTX)

Figure S3 **Changes in cell interaction with fibronectin after treatment with dasatinib at high concetrations.** JURL-MK1 (A) and HEL (B) cells (6×10^4^ per well) were seeded into fibronectin-coated E-plates. After stabilization of the microimpedance signal, 10 µM dasatinib (red circles) was added in triplets. Time of addition is indicated by an arrow. Black circles: control cells treated with 0.1% DMSO. The graphs show means and standard deviations of the triplets. Microimpedance signal (cell index) was normalized to 1 at the time of inhibitor addition.
(PPTX)

Figure S4 **Flow-cytometric analysis of SFK phosphorylation.** Cells were incubated for 2 h with imatinib or dasatinib at different concentrations, fixed and stained with anti-pSFK(Tyr416) antibody and secondary PE-anti-rabbit antibody. Mean fluorescence intensity (MFI) was measured using BD LSR Fortessa flow-cytometer and normalized to the value from the corresponding untreated control. The graphs show summary values from all independent experiments.
(PPTX)

Figure S5 **Effect of dasatinib on phospho-SFK signal in microscopic preparations.** MOLM-7 cells were plated on fibronectin-coated slide, incubated for 30 min at 37°C and treated for additional 30 min with 2 nM or 100 nM dasatinib or with 10 µM PP2.
(PDF)

Figure S6 **Western blot analysis of BCR-ABL dephosphorylation after treatment of JURL-MK1 cells with PP2.** JURL-MK1 cells were treated with PP2 at the indicated concentrations for 2 h, lysed and the level of phosphorylated BCR-ABL was assessed using anti-phospho-ABL antibody. Then the cells were fixed and stained with anti-phospho-SFK antibody (top images). Green: SFK, red: actin (stained with phalloidin), blue: nuclei (DAPI). Bottom images represent the same visual field in differential interferential contrast mode (DIC). Representative images are shown for each condition.
(PDF)
