# Correction: Real-Time Analysis of Imatinib- and Dasatinib-Induced Effects on Chronic Myelogenous Leukemia Cell Interaction with Fibronectin

**DOI:** 10.1371/journal.pone.0110070

**Published:** 2014-09-30

**Authors:** The PLOS ONE Staff

Figure 2 was incorrectly published as a duplicate of Figure S2. Please see the correct Figure 2 here.

**Figure 2:**
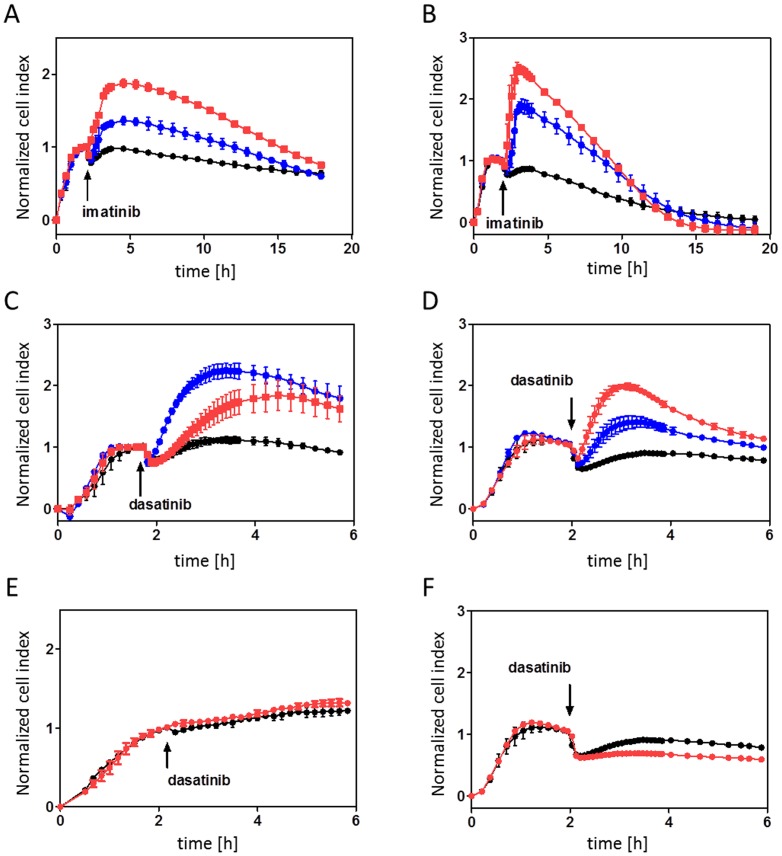
Changes in cell interaction with fibronectin after inhibitor treatment. The cells (6×10^4^ per well) were seeded into fibronectin-coated E-plates. After the microimpedance signal stabilization, the appropriate inhibitor was added in triplets. Black circles: control cells. Time of inhibitor addition is indicated by an arrow. Microimpedance signal (cell index) was normalized to 1 at the time of inhibitor addition. The graphs show mean and standard deviation of well triplets. A,C,E: JURL-MK1 cells, B,D,F: MOLM-7 cells. A,B: imatinib was added at 1 µM (blue circles) or 10 µM (red squares) final concentration. C,D: dasatinib was added at 2 nM (blue circles) or 10 nM (red squares) final concentration. E,F: dasatinib was added at 100 nM final concentration (red circles).
