# Supplementary figures and images for: Real-Time Analysis of Imatinib- and Dasatinib-Induced Effects on Chronic Myelogenous Leukemia Cell Interaction with Fibronectin
Source: PLoS One. 2014 Sep 8;9(9):e107367. doi: 10.1371/journal.pone.0107367 (PMC4157868; doi:10.1371/journal.pone.0107367)

Figure S1

A:

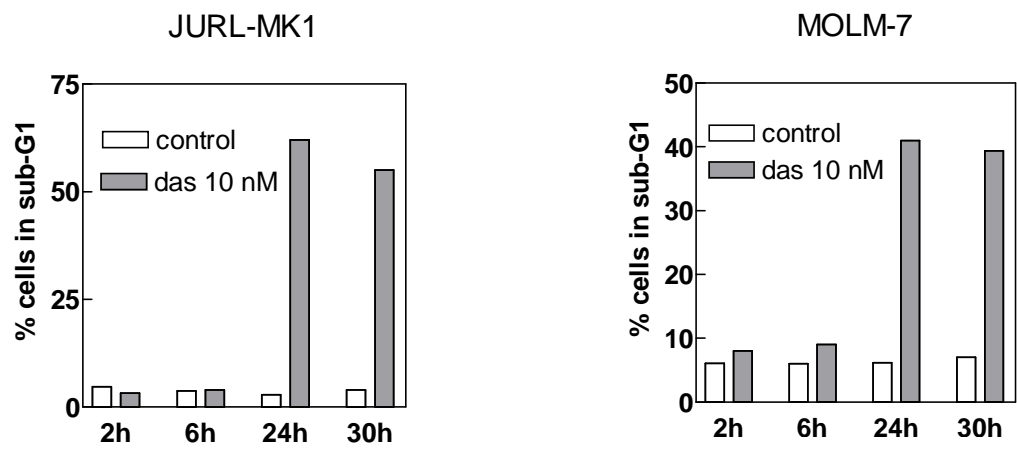

B:

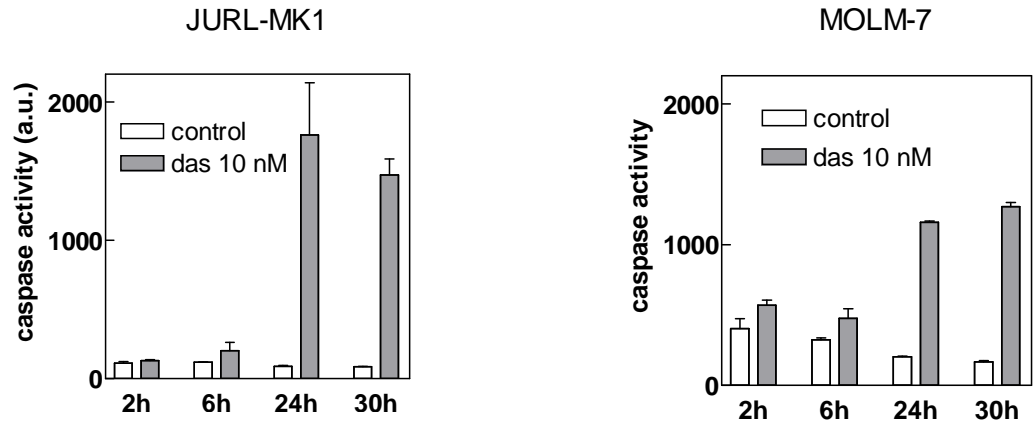

C:

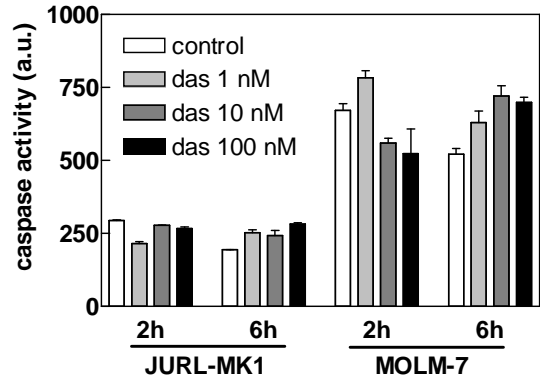

D:

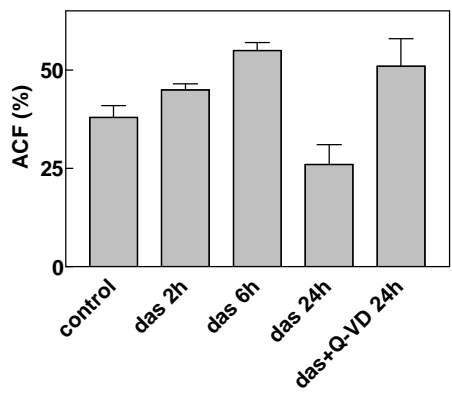

Supplement: Figure S1 — Kinetics of dasatinib-induced cell death and changes in cell adhesivity to fibronectin. A: Apoptosis induction in JURL-MK1 and MOLM-7 cells during treatment with 10 nM dasatinib was assessed by flow-cytometry. Cell fraction in sub-G1 region of cell cycle phase distribution in samples stained with propidium iodide. B: Caspase activation during cell incubation with 10 nM dasatinib was monitored using fluorogenic substrate Ac-DEVD-AFC following the same protocol as in the reference 12. C: Caspase activation in cells treated with 1 to 100 nM dasatinib for 2 or 6 h (same method as in B). D: Kinetics of changes in JURL-MK1 cell adhesivity to fibronectin following treatment with 2 nM dasatinib alone or in combination with 10 µM Q-VD-OPh (method as in Fig. 3). (PDF) [file pone.0107367.s001.pdf]

## Slide 1
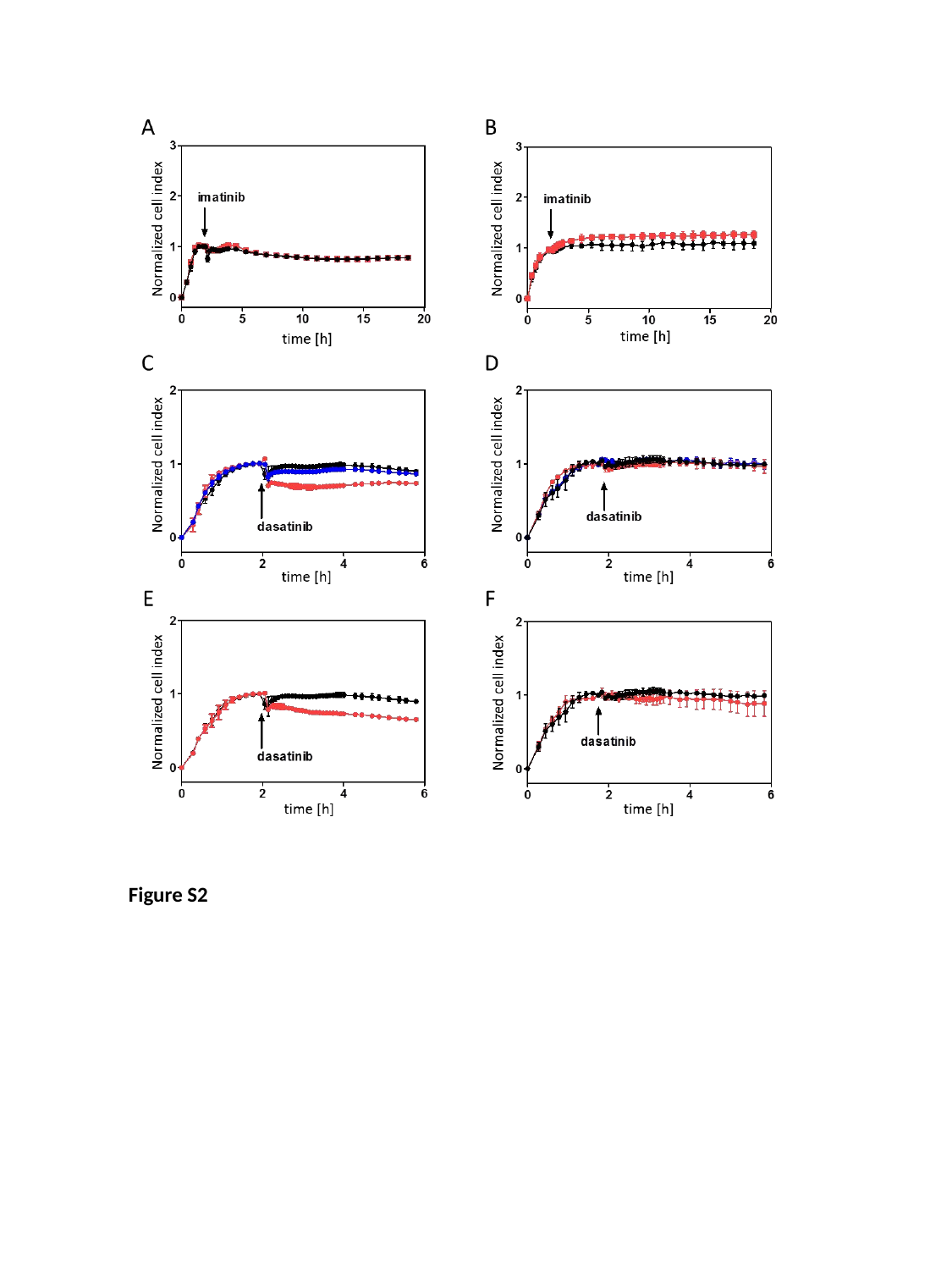

Figure S2

Supplement: Figure S2 — Changes in cell interaction with fibronectin after imatinib treatment. The cells (6×10^4^ per well) were seeded into fibronectin-coated E-plates. After the microimpedance signal stabilization, the appropriate inhibitor was added in triplets. Black circles: control cells. Time of inhibitor addition is indicated by an arrow. Microimpedance signal (cell index) was normalized to 1 at the time of inhibitor addition. The graphs show means and standard deviations of well triplets. A,C,E: HEL cells, B,D,F: JURKAT cells. A,B: imatinib was added at 10 µM (red squares) final concentration. C,D: dasatinib was added at 2 nM (blue circles) or 10 nM (red squares) final concentration. E,F: dasatinib was added at 100 nM final concentration (red circles). (PPTX) [file pone.0107367.s002.pptx]

## Slide 1
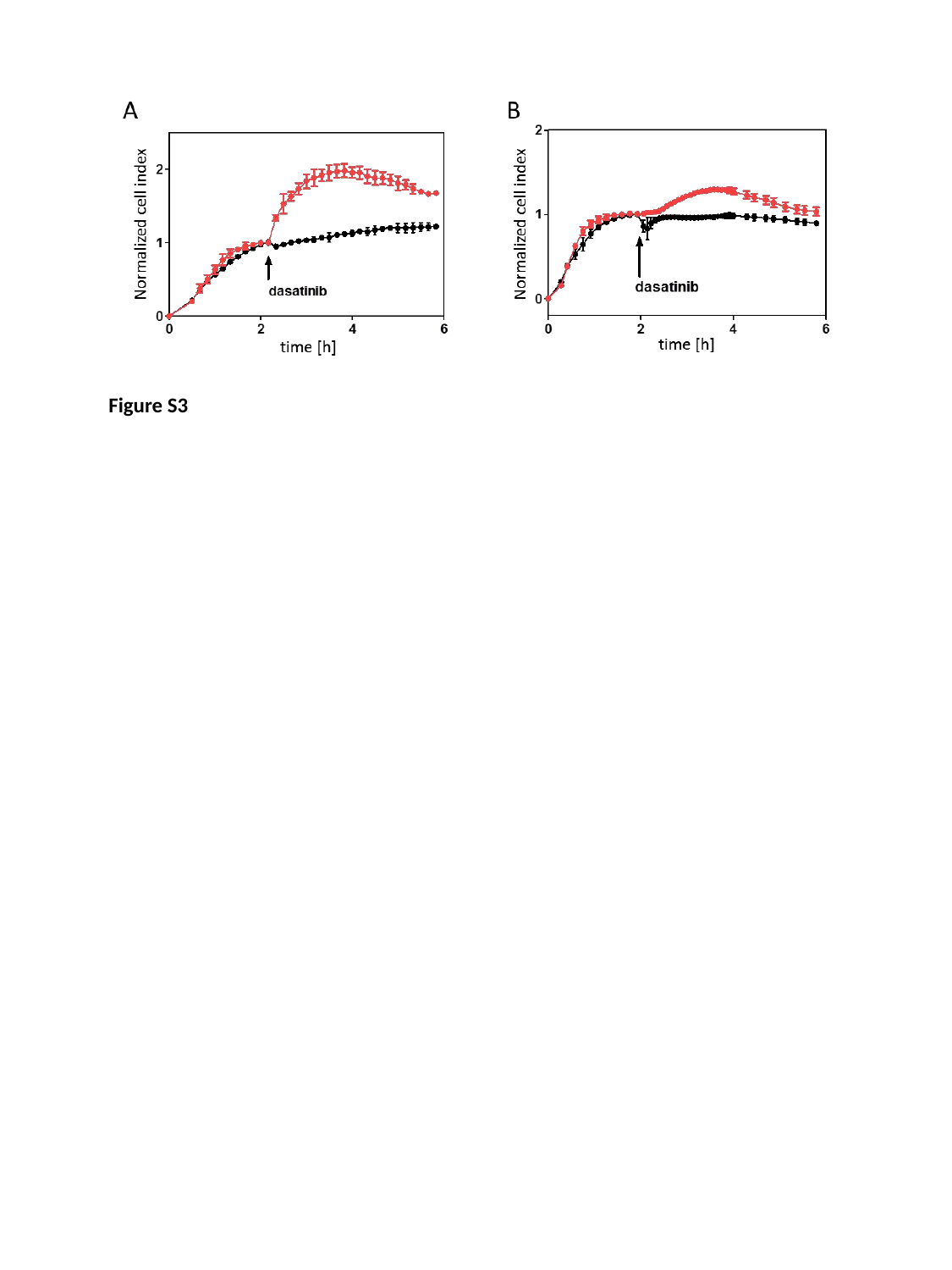

Figure S3

Supplement: Figure S3 — Changes in cell interaction with fibronectin after treatment with dasatinib at high concetrations. JURL-MK1 (A) and HEL (B) cells (6×10^4^ per well) were seeded into fibronectin-coated E-plates. After stabilization of the microimpedance signal, 10 µM dasatinib (red circles) was added in triplets. Time of addition is indicated by an arrow. Black circles: control cells treated with 0.1% DMSO. The graphs show means and standard deviations of the triplets. Microimpedance signal (cell index) was normalized to 1 at the time of inhibitor addition. (PPTX) [file pone.0107367.s003.pptx]

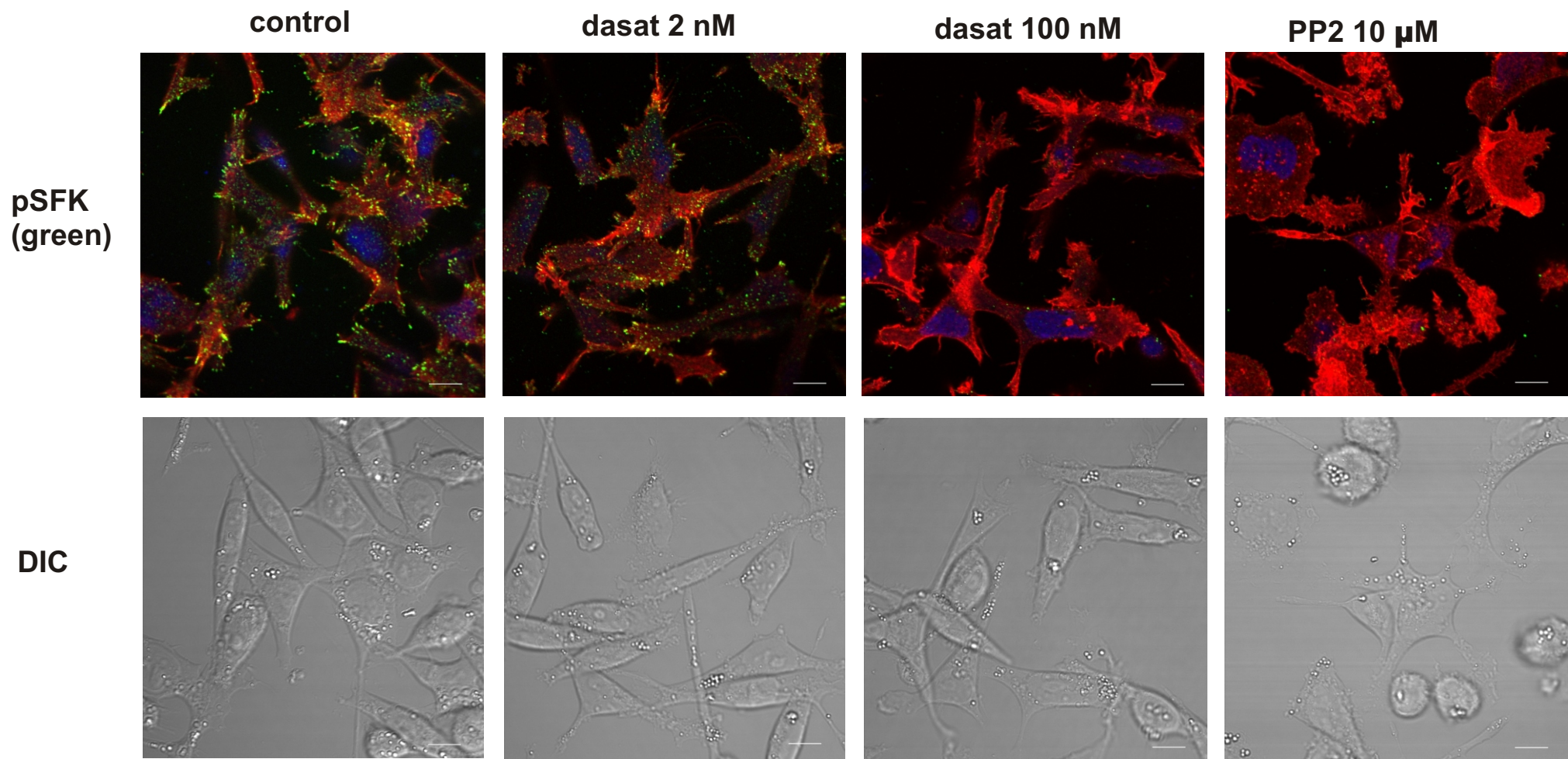

Figure S5

Supplement: Figure S5 — Effect of dasatinib on phospho-SFK signal in microscopic preparations. MOLM-7 cells were plated on fibronectin-coated slide, incubated for 30 min at 37°C and treated for additional 30 min with 2 nM or 100 nM dasatinib or with 10 µM PP2. (PDF) [file pone.0107367.s005.pdf]

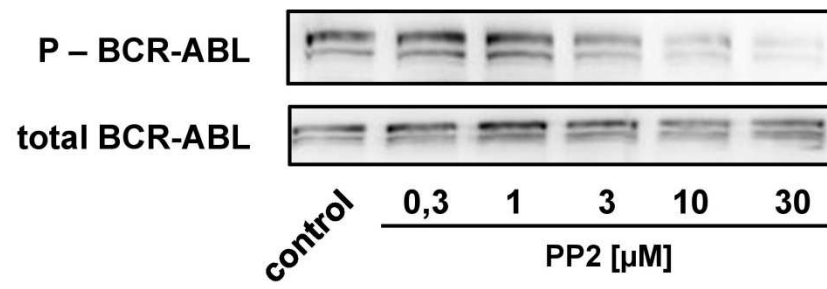

Figure S6

Supplement: Figure S6 — Western blot analysis of BCR-ABL dephosphorylation after treatment of JURL-MK1 cells with PP2. JURL-MK1 cells were treated with PP2 at the indicated concentrations for 2 h, lysed and the level of phosphorylated BCR-ABL was assessed using anti-phospho-ABL antibody. Then the cells were fixed and stained with anti-phospho-SFK antibody (top images). Green: SFK, red: actin (stained with phalloidin), blue: nuclei (DAPI). Bottom images represent the same visual field in differential interferential contrast mode (DIC). Representative images are shown for each condition. (PDF) [file pone.0107367.s006.pdf]
