# Supplementary material for: Real-Time Analysis of Imatinib- and Dasatinib-Induced Effects on Chronic Myelogenous Leukemia Cell Interaction with Fibronectin
Source: PLoS One. 2014 Sep 8;9(9):e107367. doi: 10.1371/journal.pone.0107367 (PMC4157868; doi:10.1371/journal.pone.0107367)
Supplement: Figure S4 — Flow-cytometric analysis of SFK phosphorylation. Cells were incubated for 2 h with imatinib or dasatinib at different concentrations, fixed and stained with anti-pSFK(Tyr416) antibody and secondary PE-anti-rabbit antibody. Mean fluorescence intensity (MFI) was measured using BD LSR Fortessa flow-cytometer and normalized to the value from the corresponding untreated control. The graphs show summary values from all independent experiments. (PPTX) [file pone.0107367.s004.pptx]

## Slide 1
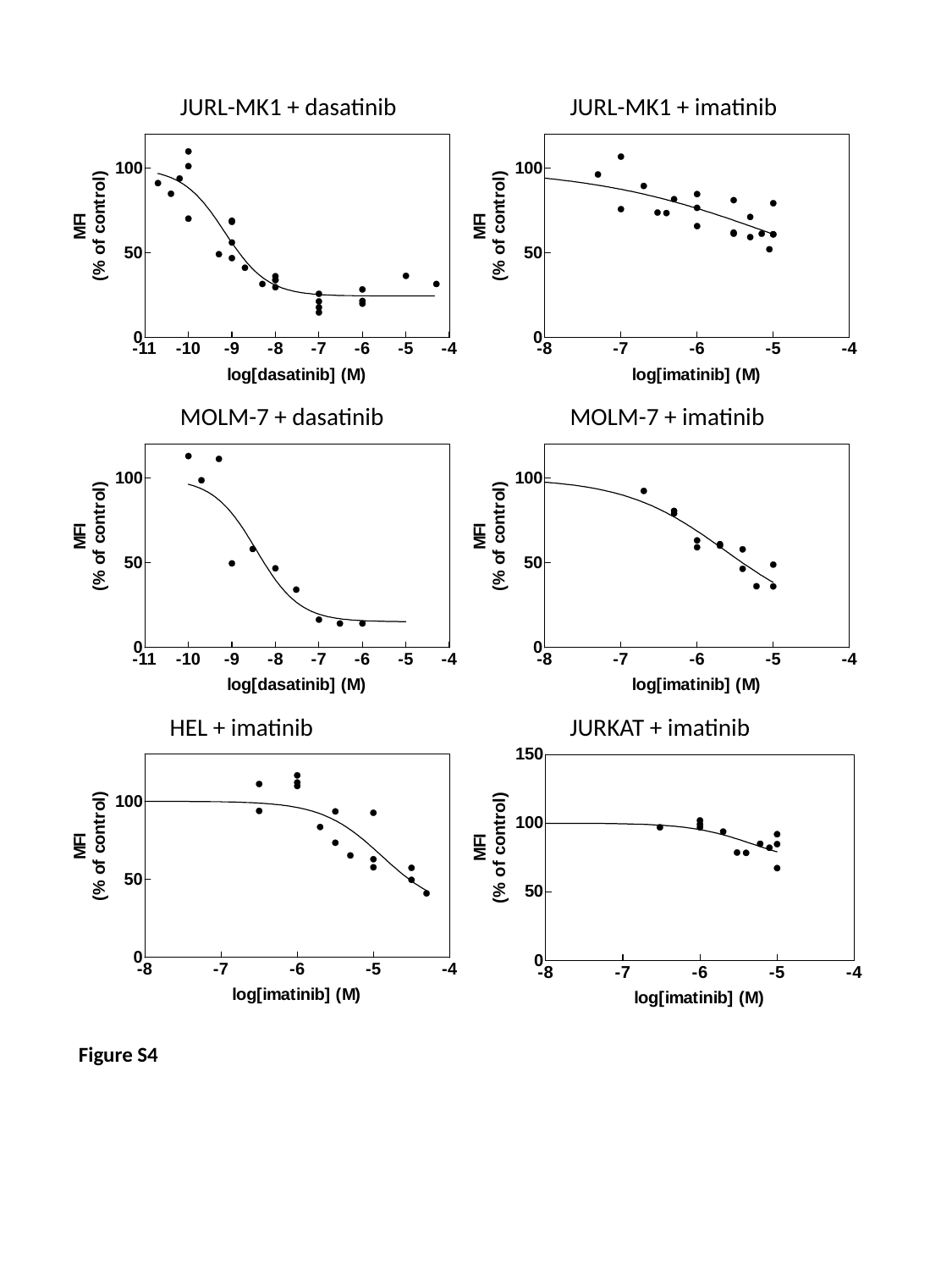

JURL-MK1 + dasatinib
JURL-MK1 + imatinib
MOLM-7 + dasatinib
MOLM-7 + imatinib
HEL + imatinib
JURKAT + imatinib
Figure S4
